# Flax transposons: unraveling their impact on domestication and agronomic trait variation

**DOI:** 10.18699/vjgb-25-131

**Published:** 2025-12

**Authors:** М.A. Duk, V.A. Stanin, A.A. Kanapin, А.A. Samsonova, Т.A. Rozhmina, М.G. Samsonova

**Affiliations:** Ioffe Institute of the Russian Academy of Sciences, St. Petersburg, Russia; Peter the Great St. Petersburg Polytechnic University, St. Petersburg, Russia; Peter the Great St. Petersburg Polytechnic University, St. Petersburg, Russia; Peter the Great St. Petersburg Polytechnic University, St. Petersburg, Russia; Federal Research Center for Bast Fiber Crops, Torzhok, Russia; Peter the Great St. Petersburg Polytechnic University, St. Petersburg, Russia

**Keywords:** flax, Linum usitatissimum, transposons, GWAS, genetic diversity, breeding, лен, Linum usitatissimum, транспозоны, GWAS, генетическое разнообразие, селекция

## Abstract

Flax is an important agricultural crop with multifunctional uses. Diversified breeding for oil content in seeds and fiber in stems has led to the emergence of two morphotypes – fiber flax and oilseed flax. Previously, using single nucleotide polymorphisms (SNPs), we characterized the genetic diversity of 306 flax samples from the collection of the Russian Federal Research Center for Bast Crops. However, larger structural variations, such as mobile genetic elements, also play a significant role in shaping agronomically important plant traits and can be used for further flax improvement. Here, we used the same flax collection to predict sites of new transposon insertions and to assess the role of such insertions in the formation of agronomically important traits, as well as in the process of flax domestication. We discovered 588,480 new transposon insertion sites not present in the reference flax genome (NCBI assembly ASM22429v2), the majority of which were attributed to retrotransposons of the Copia and Gypsy superfamilies, while among DNA transposons, insertion sites of the MULE-MuDR, hAT, and CMC-EnSpm superfamilies were most common. Unlike SNPs, which were significantly more numerous in oilseed flax than in fiber flax, we did not find such a substantial difference in the number of insertions of different transposon families per plant among samples of different morphotypes. Analysis of genomic regions affected by recent breeding efforts revealed a total of 61 candidate regions, of which 18 regions overlapped with QTLs associated with important agronomic traits. Interestingly, 5 regions of reduced genetic diversity in kryazhs and cultivars compared to landraces were also identified as regions of reduced diversity when using single nucleotide polymorphisms as markers. A genome-wide association study (GWAS) identified 50 TE insertions associated with different phenotypic traits, with many associations confirmed by multiple models or detected in data from multiple years. Thus, transposon insertion sites are an important source of genetic diversity in flax, alongside single nucleotide polymorphisms, making them suitable for further crop improvement in breeding.

## Abstract (ru)

Транспозоны льна: роль в генетическом разнообразии, окультуривании и детерминации хозяйственно ценных признаков

## Introduction

Flax is an important agricultural crop grown for both fiber and oil, used in many areas such as the production of varnishes and paints, linoleum, composites, and the textile and food industries (Goudenhooft et al., 2019). Long-term breeding of flax for oil content in seeds and fiber in stems has led to the appearance of two morphotypes – fiber flax and oilseed flax. Fiber flax is characterized by less branching, greater stem length and plant height, while oilseed flax is characterized by a larger number of seeds, and hence a greater number of inflorescences, with a shorter main stem length. In the late 19th and early 20th centuries, Russia was the main supplier of high-quality flax fiber, obtained from Russian heritage landraces, also known as “kryazh” (plural: kryazhs) resulting from folk selection. Kryazhs and Russian landraces made a decisive contribution to the gene pool of modern flax cultivars (Helbaek, 1959; Duk et al., 2021).

Previously, using single nucleotide polymorphisms, we characterized the genetic diversity of 306 flax samples from the collection of the Russian Federal Research Center for Bast Crops (FRC BC). We found significant differentiation between oilseed and fiber flax populations and identified genomic regions affected by modern breeding (Kanapin et al., 2022; Duk et al., 2025).

However, larger structural variations, such as transposon insertions (TEs), also play a significant role in shaping agronomically important plant traits and can be used for further improvement of the flax cultivars. It is known that TEs constitute a large part of plant genomes (Quesneville, 2020), and their insertions can lead to genome rearrangements, gene silencing, and rewiring of gene networks (Bourque et al., 2018), and can also be a source for the emergence of new genes and non-coding RNAs (Pulido, Casacuberta, 2023).

TEs are conventionally divided into two classes. Class I includes retrotransposons, which increase their copy number in the genome by insertion via an RNA intermediate (Mhiri et al., 2022), resulting in long terminal repeats potentially constituting up to 90 % of a plant’s genome. Class II includes DNA transposons, which operate on a “cut-and-paste” principle, moving around the genome and usually not increasing their copy number. The highest TE activity is observed during periods of stress (Schrader, Schmitz, 2019). Most often, new insertions have a negative effect and are removed from the population; however, sometimes they can promote plant adaptation to unfavorable environmental conditions (Niu et al., 2019) and, because of this, be preserved in the population as a result of positive selection.

In this work, we used the same collection of 306 flax samples from the FRC BC (Duk et al., 2021; Kanapin et al., 2022) to predict sites of new TE insertions in groups of samples of different morphotypes and breeding status and to compare the distribution patterns of TE insertion sites and polymorphic sites across the genome. We also assessed the role of TE insertions in the formation of agronomically important traits and in the process of flax domestication

## Materials and methods

A total of 306 flax samples from the Federal Research Center for Bast Crops (FRC BC, Torzhok, Russia) collection were used in this study. The panel comprised 182 fiber flax and 120 oilseed flax varieties. The oilseed group included 99 intermediate, 16 crown, and five large-seeded accessions; the morphotype of the four remaining accessions was undetermined. Based on breeding status, the accessions were categorized as follows: 230 cultivated varieties (including 141 cultivars and 89 breeding lines), 40 landraces, and 31 kryazhs

Genomic DNA was extracted from leaf tissue using the DNeasy Plant Mini Kit (Qiagen, Netherlands). Library preparation and sequencing were performed at BGI on an Illumina platform, generating 150 bp paired-end reads. This yielded approximately 7.63 billion raw reads, totaling 1,143.850625 Gb of data. The average genome coverage was 9.3×, corresponding to 3.7 Gb per sample.

TE insertion sites were predicted using the TEMP2 software package (Yu T. et al., 2021) in insertion2 mode to identify non-reference insertions. Consensus TE sequences for the search were generated de novo with the REPET package’s TEdenovo module (Flutre et al., 2011). To address inherent imprecision in insertion coordinates, we clustered insertions of the same TE family that were within 200 bp of each other (twice the sequencing fragment length) and assigned them a unified coordinate at the center of the cluster. The final calls were converted to VCF format, and key insertions were visually validated using the Integrative Genomics Viewer (IGV).

For population and GWAS analyses, we further consolidated insertion calls to minimize false positives. Insertions across accessions located within a 50 bp window were merged into a single locus, with the position defined as the window’s midpoint. This 50 bp threshold was empirically chosen as it maximized the number of insertions with a minor allele frequency (MAF) >5 %, thereby increasing statistical power while reducing the likelihood of spurious associations.

The genetic structure in the dataset was evaluated using the Principal Component Analysis (PCA) as well as the ADMIXTURE software v.1.3.0 (Alexander et al., 2009). The analyses were performed for K values ranging from 2 to 5. The phylogenetic tree was constructed with the ape package and drawn using the ggtree R package (Yu G., 2020). To assess genetic diversity due to TE insertions and calculate the fixation index (Fst), the VCFtools tool (Danecek et al., 2011) was used, with a window size of 200 kb. The window size was chosen in accordance with the average size of regions found as “hotspots” in the PrimatR package for R (https://github. com/daewoooo/primatR).

To identify possible genomic regions that underwent selection during breeding, we compared cultivars with the corresponding kryazhs and landraces. In each comparison, we calculated Fst and ROD = 1 – π1/π2 statistics, where π is the genetic diversity of the corresponding sample group. Genomic regions with high population differences between the two groups (highest Fst values, top 5 % of the entire genome and top 2.5 % of ROD values) were considered as possible regions with traces of selection.

Values of 13 phenotypic traits measured in plants grown on the experimental fields of FRC BC in 2019 (one cultivation), 2020 (two cultivations with a two-week shift), and 2021 (one cultivation) were used (Kanapin et al., 2022): DSI – fusarium wilt severity index, EFL – elementary fiber length, FC – fiber content, FW – fiber weight, IL – inflorescence length, NI – number of internodes, Oil – oil content in seeds, PH – plant height, Nsed – number of seeds per plant, STI – stem tapering index, TL – technical stem length, TW – weight of the technical part of the plant, Tswgt – 1,000-seed weight. The genome-wide association analysis was performed using Blink, FarmCPU, SUPER, MLMM, MLM, GLM models in the GAPIT package (Wang, Zhang, 2020) with a threshold FDR rate 0.9. To link associations with genes, the genome annotation provided by the S. Cloutier group (You, Cloutier, 2020) was used. To calculate the effect of the insertion on the trait, the trait values in samples with and without the insertion were compared, with reliability confirmed by the Mann–Whitney statistical test (Mann, Whitney, 1947).

## Results

**Composition of the flax mobilome**

**Fig. 1.**
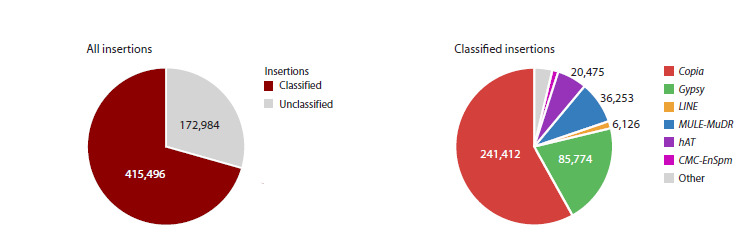
Total number of new TE insertions and the number of insertions of individual TE superfamilies in the collection samples

We identified a total of 588,480 new transposable element (TE) insertions across 306 flax samples, 172,984 (29.4 %) of which could not be classified (Fig. 1). Among the classified insertions, the Copia superfamily was predominant, representing 41 % of all insertions (58 % of classified insertions). The Gypsy superfamily was the next most abundant, comprising 15 % of all insertions (20 % of classified ones). Retrotransposons of the LINE superfamily constituted 1 % of insertions. Among Class II DNA transposons, the Mutator (MULE-MuDR) superfamily was the most common (6 % of all insertions), followed by the hAT (4.3 %) and CMC-EnSpm (1.3 % of classified insertions) superfamilies.

Population structure analysis using PCA indicated that genetic differentiation between fiber and oilseed morphotypes was primarily driven by insertions from the Copia and Gypsy superfamilies, as well as the hAT-Ac family, as these were the only markers for which the first principal component separated the two groups (Fig. S1)1.

Supplementary Materials are available in the online version of the paper: https://vavilov.elpub.ru/jour/manager/files/Suppl_Duk_Engl_29_8.pdf


**Genomic landscape of the flax mobilome**

**Fig. 2.**
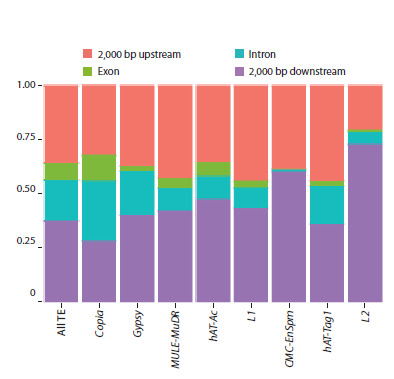
Location of TE insertion sites relative to genes

The genomic distribution of TE insertions relative to genes varied significantly among superfamilies (Fig. 2, Table S1). Overall, 22 % of all insertions were located within or in close proximity (<2 kb) to genes. A strong bias for intergenic regions was observed for the Gypsy (85 %) and CMC-EnSpm (74 %) superfamilies, with the majority of their insertions located far (>2 kb) from genes. In contrast, approximately half of all LINE and hAT insertions were found near or within genes. The Copia superfamily showed a pronounced preference for genic regions, inserting into genes 1.4 times more frequently than the overall average. Furthermore, Gypsy elements that did land within genes were 1.3 times more likely to be in introns and three times less likely to be in exons compared to the general TE population. Exonic insertions were exceptionally rare for the CMC-EnSpm and L2 transposons. Across all superfamilies, intragenic insertions were predominantly intronic (71 %), with only 29 % located in exons.

**Using TE insertion data for population structure and relatedness analyses**

**Fig. 3.**
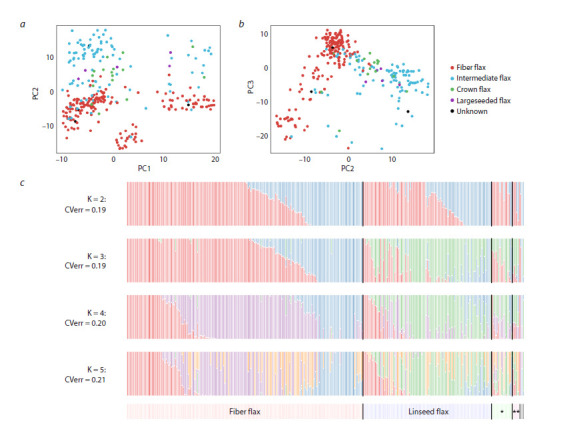
Population structure of flax accessions a, b – visualization of flax accession clustering using PCA; c – ADMIXTURE graphs for K = 2–5 populations. CVerr – cross-validation error value during analysis; * crown; ** large-seeded accessions

Principal Component Analysis revealed limited population structure based on TE insertions. While some differentiation between fiber and oilseed morphotypes was visible along the second and third principal components, no clear grouping by breeding status was observed (Fig. 3a, b). ADMIXTURE analysis indicated that the optimal number of genetic populations (K) was two, based on cross-validation error (Fig. 3c). However, the error for K = 3 was only marginally higher. At K > 2, a distinct genetic component (shown in green in Fig. 3c) became apparent specifically within oilseed flax accessions. No discernible differences in admixture patterns were associated with breeding status.

**Fig. 4.**
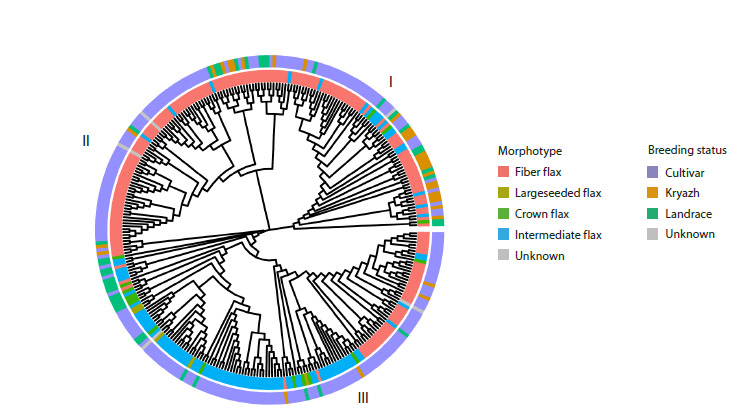
NJ tree colored by morphotype and breeding status

Phylogenetic reconstruction supported the population structure, grouping the accessions into three distinct clades (Fig. 4). Clades I and II were composed almost exclusively of fiber flax, while Clade III contained nearly all oilseed flax samples

Kryazhs were predominantly found in Clade I, confirming their shared genetic background (Duk et al., 2021). Each clade was significantly enriched for specific TE insertions. Clade I was characterized by ten unique TE sequences, primarily from the Copia (five) and Gypsy (two) superfamilies, with single sequences from RC/Helitron and hAT-Ac. Clades II and III were enriched with two and three Copia superfamily insertions, respectively (Table S2).

**Identifying selective sweeps and analysis of agronomic traits using transposon insertions**

**Fig. 5.**
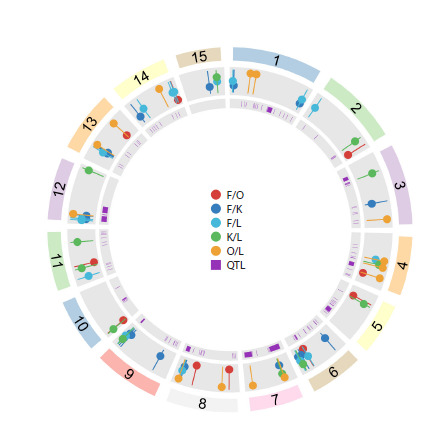
Genomic regions under selection. Circos diagram showing genome- wide selective sweep loci identified in different comparisons: fiber flax vs. oilseed flax (F/O), fiber flax cultivars vs. landraces (F/L), fiber flax cultivars vs. kryazhs (F/K), kryazhs vs. fiber flax landraces (K/L), oilseed flax cultivars vs. landraces (O/L). QTL – QTLs published in (You, Cloutier, 2020).

To uncover genomic signatures of selection, we scanned for regions exhibiting significantly reduced diversity in specific comparisons: a) fiber flax cultivars vs. kryazhs or landraces; b) landraces vs. kryazhs; and c) oilseed cultivars vs. oilseed landraces. We also compared genetic diversity between fiber and oilseed cultivars to identify regions associated with their divergent agronomic traits. This analysis identified 61 candidate selective sweep regions (Fig. 5), with nine regions detected in multiple comparisons. Notably, 18 of these candidate regions overlap with known quantitative trait loci (QTLs) for important agronomic traits (You, Cloutier, 2020) (Table S3), linking these signatures of selection to specific phenotypic outcomes.

Comparative genomic analysis revealed distinct selective sweep patterns between flax morphotypes and breeding groups, with many overlapping known quantitative trait loci (QTLs). The comparison between oilseed and fiber flax revealed only one selective sweep signal in fiber flax, which co-localizes with the oil content QTL QOIL-Lu6.4 on chromosome 6 (Table S3). Cultivated varieties of both morphotypes showed reduced diversity (as compared to landraces) in regions of chromosome 12 overlapping with QTLs QIODLu12.3, QLIN-Lu12.3, QLIO-Lu12.3, associated with iodine content, linoleic and linolenic acid content, respectively.In fiber flax cultivars, selective sweep signals were observed 1) in a region of chromosome 12, overlapping with QTL uq.C12-1, 2) in a region of chromosome 3 overlapping with Lu3-25559600, 3) in a region of chromosome 8 overlapping with QOLE-Lu8.1, 4) in a region of chromosome 9 overlapping with QSTE-Lu9.2, and 5) in regions of chromosome 6 overlapping with Lu2564 and QOIL-Lu6. These QTLs are associated with plant height and stem length, seed mucilage content, oleic and stearic acid content, and oil content, respectively.

In kryazhs, selective sweep signals were detected in 1) regions of chromosome 7 overlapping with QPM-crc-LG7 and QPAL-Lu7.3, which are associated with powdery mildew incidence and palmitic acid content, respectively; 2) in regions of chromosome 2 overlapping with scaffold43-1111162 and QOIL-Lu2.1, which are associated with 1,000-seed weight and oil content, respectively, and 3) in regions of chromosome 3 overlapping with QLio-LG3.1, QLin-LG3.1, Marker4371 and scaffold156-76129, for which association with linolenic and linoleic acid content, plant height, and number of bolls, respectively, has been shown.

In oilseed flax cultivars, reduced diversity was also observed in regions of chromosome 8 overlapping with scaffold635- 4397 and QOLE-Lu8.1, which are associated with the number of branches and oleic acid content, respectively; conversely, increased diversity compared to landraces was observed in regions of chromosome 7 overlapping with QLIN-Lu7.2, QLIO-Lu7.2, QPAL-Lu7.3 and QIOD-Lu7.2, which are associated with linolenic, linoleic, palmitic acid, and iodine content, respectively.

To further elucidate the genetic basis of agronomic traits, we conducted a GWAS utilizing transposon insertions as molecular markers. We discovered 50 TE insertions significantly associated with traits such as Fusarium wilt resistance, productivity, and fiber content. Many of these associations were robust, being confirmed by multiple models or repeated across growing seasons. A ~20 % subset exhibited pleiotropic effects, associating with several traits simultaneously (see the Table and Table S4). The potential functional importance of these insertions is underscored by the finding that four reside within known QTLs and two are located in genomic regions with significantly reduced diversity (Fig. 5).

Specifically, 15 associations were supported by multiple models, and 12 were linked to multiple traits or years. Six widely distributed insertions (found in >50 accessions) were selected for experimental validation, with the results detailed in the Figures S2–S4.

## Discussion

A detailed characterization of flax genetic diversity is of paramount importance for its long-term and sustainable production and diversification, as well as for the overall success of its breeding programs. Previously, using SNPs, we characterized the genetic diversity of the core flax collection (306 samples) of the Federal Research Center for Bast Crops (FRC BC) (Duk et al., 2021; Kanapin et al., 2022). This collection, one of the best in the world, includes flax varieties from Eurasia with a significant proportion of local varieties. Recently, thanks to advances in bioinformatics and the improvement of sequencing technologies, other sources of genomic diversity, including TEs and structural variation, have become available for analysis, which can play a significant role in shaping agronomically important plant traits and can be used for further improvement of agricultural crops.

Analysis of new TE insertion sites performed in this work showed that, along with SNPs, TEs are an important source of genetic diversity in flax. The predominant insertion sites, as in many other agricultural plants (Stanin et al., 2025), are retrotransposon insertions, and among DNA transposons, insertions of the MULE-MuDR, hAT, and CMC-EnSpm superfamilies are most common (Fig. 1).

In contrast to SNP variation, which was greater in oilseed flax and landraces than in fiber flax, the number of TE family insertions did not differ substantially between morphotypes. Despite this, TE insertion patterns still corroborate the close relationship and common origin of the kryazhs, similarly to SNPs. Furthermore, the insertion profiles of the Copia, Gypsy, and hAT-Ac superfamilies clearly distinguish oilseed from fiber flax varieties (Fig. S1).

The genomic location of TEs relative to genes significantly influences gene expression and can lead to diverse phenotypic changes. In flax, most Gypsy and CMC-EnSpm insertions are located in intergenic regions. Furthermore, among the TEs that have inserted into genes, only 29 % are found in exons, suggesting that insertions in these coding regions are preferentially purged by natural selection (Fig. 2).

Analysis of genomic regions impacted by recent breeding (Table S3) revealed a striking disparity in selection signals between oilseed and fiber flax cultivars, with 9 and 32 regions identified, respectively. Notably, chromosomes 2, 5, 6, 9–11, and 15 showed no signals of selective improvement in oilseed flax (Fig. 5). Despite the fewer regions in oilseed flax, two of them overlap with known QTLs for fatty acid synthesis. We also identified 10 genomic regions showing divergent selection between the two morphotypes. A comparison of fiber flax cultivars and kryazhs revealed numerous selective sweep regions, but these showed little overlap. This likely reflects differing breeding objectives for modern fiber flax, driven by new industrial uses and climate change. This hypothesis is supported by the detection of 13 regions with reduced diversity in modern fiber cultivars compared to kryazhs.Interestingly, five regions showing reduced genetic diversity in cultivars and kryazhs compared to landraces are also identified as regions of the diversity reduction when SNPs are used as markers (Table S5). Among these, noteworthy is the region of reduced diversity in cultivated oil flax varieties within Chr4_12400001–12600000, which contains the gene Lus10036915 associated with pathogen defense, as well as the region Chr8_22400001–22600000, which overlaps with QTLs scaffold_6354_3971 and QOLELu8.1, associated with branch number and oleic acid content, respectively.

The most robust association identified in our GWAS was for the TE insertion Chr4_14756320, which was significantly linked to 22 distinct trait-year combinations using all analytical models (see the Table). This variant is a hAT-Ac family insertion situated ~2 kb upstream of Lus10041548, a gene with an AGAMOUS-like ortholog function that controls meristem determinacy and floral development (Yanofsky et al., 1990). The insertion’s association with increased inflorescence length but decreased fiber quality suggests it may inhibit fiber cell initiation in the meristem. Its genomic position overlaps with three key QTLs: QYLD-Lu4.1 (seed yield), QPLH-Lu4.3 (plant height), and QDTM-Lu4.1 (early maturity).

**Table 1.**
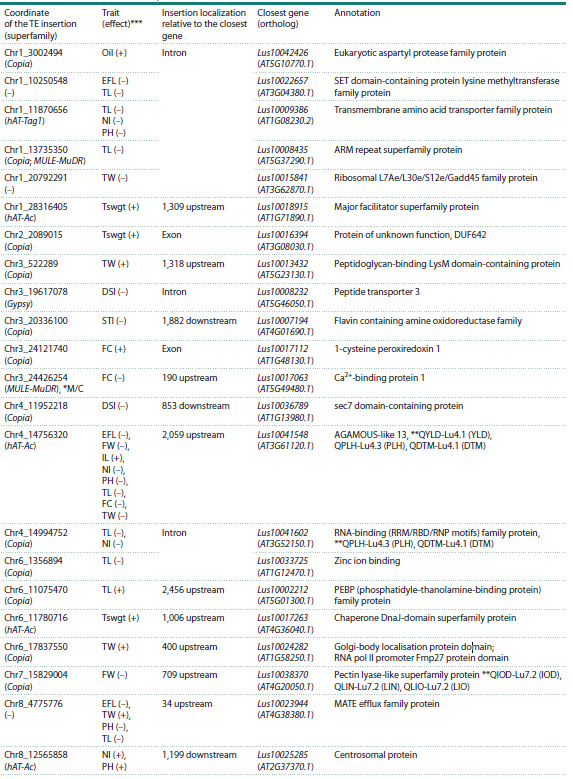
TE insertions associated with phenotypic traits and located less than 3,500 bp from genes with known function * Insertion falls within a genomic region under selection identified in different comparisons: fiber flax vs. oilseed flax (F/O), oilseed flax cultivars vs. landraces (O/L). ** Insertion also falls within a QTL published in (You, Cloutier, 2020). TE insertion coordinate is the midpoint of a 50 bp window. *** Effect of the TE insertion on the trait: (+) – positive, (–) – negative

**Table 1end.**
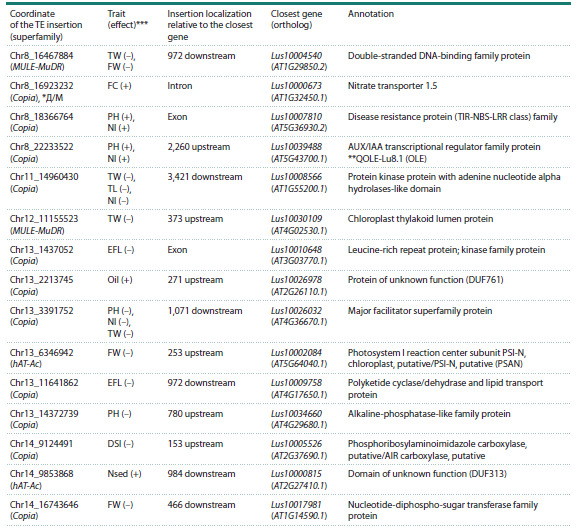
Table 1end.

We also noted that the same phenotypic effect could be caused by different TE families inserting at identical sites, such as Chr1_13735350 (Copia/MULE-MuDR) and Chr13_2207778 (MULE-MuDR/hAT-Tag1).Contrary to expectation, several exonic insertions appear to enhance trait performance. For instance, Copia insertions in the exon of Lus10016394, Lus10017112, and Lus10007810 were associated with positive effects, indicating that these genes likely function as suppressors of their respective traits (see the Table).

We identified pleiotropic TE insertions affecting multiple plant architecture traits. An intronic hAT-Tag1 insertion in Lus10009386 (Chr1_11870656), a gene involved in amino acid transport (Meyer et al., 2006), negatively impacted technical stem length, plant height, and internode number. A comparable negative effect on stem and internode development was caused by an intronic Copia insertion in Lus10041602 (Chr4_14994752), a gene encoding a photosynthetic apparatus component with a role in germination stress response (Xu et al., 2013); this locus also overlaps with QTLs for plant height and early maturity (see the Table).

Conversely, a Copia insertion upstream of Lus10039488 (Chr8_22233522) enhanced plant height and internode number. This gene modulates early auxin responses (Liscum, Reed, 2002), and the insertion lies within the QOLE-Lu8.1 QTL associated with oleic acid.

 The TE insertion at Chr1_10250548 located in an intron of the Lus10022657 gene, whose ortholog contributes to the transcriptional suppression of pseudogenes and transposons (Veiseth et al., 2011), had a negative effect on technical stem length and fiber length (see the Table).

Some insertions can also have different effects on different traits. For example, the insertion Chr8_4775776, located upstream of Lus10023944, had a negative effect on fiber length but a positive effect on the weight of the technical part. Such an influence is more preferable when it comes to oil flax, and the insertion was likely preserved during the selection process. The ortholog of this gene belongs to the MATE protein family, which is involved in protection from toxins and the synthesis of beneficial compounds (Takanashi et al., 2014).

The statistical robustness of several associations was confirmed by their discovery with multiple analytical models. A notable example is a Copia insertion at Chr1_3002494, located within an intron of the Lus10042426 gene, which was associated with increased oil content. The ortholog of this gene is implicated in plant immunity (Breitenbach et al., 2014). Other robust associations include intronic Copia insertions at Chr6_1356894 and Chr8_16923232. The corresponding genes are involved in root development metabolism (Takemoto et al., 2018) and nitrate transport (Lin et al., 2008), respectively. Furthermore, the insertion at Chr8_16923232 is located within a selective sweep region that differentiates oilseed flax from fiber flax (see the Table).

From an applied perspective, TE insertions that confer advantageous traits can be harnessed in breeding. A compelling case is the association between reduced disease severity (DSI) and two insertions: a Gypsy element in the 3′-flanking region of Lus10008232 (implicated in seed stress resistance and pathogen defense) (Karim et al., 2005) and a Copia element within the auxin transport and cell wall organization gene Lus10036789 (Geldner et al., 2003). These variants provide direct targets for marker-assisted selection to enhance disease resistance.

## Conclusion

The genomic landscape of transposon insertions in flax is non-uniform, revealing patterns consistent with the divergent selection pressures applied to different morphotypes. These insertions contribute substantially to phenotypic diversity and environmental adaptation. Consequently, transposons serve as a crucial source of molecular markers that, together with single nucleotide polymorphisms, can be harnessed to select for desired characteristics in breeding programs.

## Conflict of interest

The authors declare no conflict of interest.
